# JAK3 as an Emerging Target for Topical Treatment of Inflammatory Skin Diseases

**DOI:** 10.1371/journal.pone.0164080

**Published:** 2016-10-06

**Authors:** Ana Karina Alves de Medeiros, Reinhart Speeckaert, Eline Desmet, Mireille Van Gele, Sofie De Schepper, Jo Lambert

**Affiliations:** 1 Department of Dermatology, Ghent University Hospital, Ghent, Belgium; 2 Department of Dermatology, Ghent University, Ghent, Belgium; University of Kansas Medical Center, UNITED STATES

## Abstract

The recent interest and elucidation of the JAK/STAT signaling pathway created new targets for the treatment of inflammatory skin diseases (ISDs). JAK inhibitors in oral and topical formulations have shown beneficial results in psoriasis and alopecia areata. Patients suffering from other ISDs might also benefit from JAK inhibition. Given the development of specific JAK inhibitors, the expression patterns of JAKs in different ISDs needs to be clarified. We aimed to analyze the expression of JAK/STAT family members in a set of prevalent ISDs: psoriasis, lichen planus (LP), cutaneous lupus erythematosus (CLE), atopic dermatitis (AD), pyoderma gangrenosum (PG) and alopecia areata (AA) versus healthy controls for (p)JAK1, (p)JAK2, (p)JAK3, (p)TYK2, pSTAT1, pSTAT2 and pSTAT3. The epidermis carried in all ISDs, except for CLE, a strong JAK3 signature. The dermal infiltrate showed a more diverse expression pattern. JAK1, JAK2 and JAK3 were significantly overexpressed in PG and AD suggesting the need for pan-JAK inhibitors. In contrast, psoriasis and LP showed only JAK1 and JAK3 upregulation, while AA and CLE were characterized by a single dermal JAK signal (pJAK3 and pJAK1, respectively). This indicates that the latter diseases may benefit from more targeted JAK inhibitors. Our *in vitro* keratinocyte psoriasis model displayed reversal of the psoriatic JAK profile following tofacitinib treatment. This direct interaction with keratinocytes may decrease the need for deep skin penetration of topical JAK inhibitors in order to exert its effects on dermal immune cells. In conclusion, these results point to the important contribution of the JAK/STAT pathway in several ISDs. Considering the epidermal JAK3 expression levels, great interest should go to the investigation of topical JAK3 inhibitors as therapeutic option of ISDs.

## Introduction

Inflammatory skin diseases (ISDs) are very prevalent worldwide and have a serious impact on the patients’ quality of life. However, treatment options remain scarce with corticosteroids being the main topical option. Recent advances on the role of cytokines in the pathophysiology of immune mediated inflammatory diseases lead to the understanding that many pro-inflammatory interleukins use JAK/STAT components for signal transduction [1, 2]. Briefly, the JAK/STAT signaling pathway transmits information from extracellular chemical signals to the nucleus resulting in DNA transcription. Binding of ligands, such as interferon and interleukins, to their specific transmembrane receptors activate associated JAKs. Subsequently, activated JAKs (Janus kinases) phosphorylate tyrosine residues on the receptor, creating docking sites for latent STATs (Signal Transducer and Activator of Transcription). After recruitment of STAT to the receptor, they are also phosphorylated by JAKs. Activated STATs migrate to the nucleus of the cell and promote gene transcription or induction [3, 4]. In mammals, the JAK/STAT family consists of 4 JAK members (JAK1, JAK2, JAK3 and TYK2) and 7 STAT members (STAT1, STAT2, STAT3, STAT4, STAT5a, STAT5b, STAT6) [3]. The JAKs are selectively activated by different receptors and have, therefore, distinct *in vivo* roles [4]. JAK1 is mainly activated by type II cytokine receptors. JAK2 is crucial in transducing signals for cytokine receptors involved in hematopoiesis (erythropoietin, thrombopoietin and haematopoietic cell development cytokines). JAK3 is mainly expressed in B and T lymphocytes, and TYK2 associates commonly with other JAKs [5].

The recent discovery of the JAK/STAT signaling pathway opened a new window of opportunity for the treatment of ISDs and promoted the development of drugs that block JAK activation [1, 2]. The kinase domain of JAKs makes them an easier pharmacological target compared to STATs, which do not have catalytic activity [3]. One of the advantages of JAK inhibitors is their structure. They are small molecules, which can easily penetrate the epidermal barrier and therefore be used in topical formulations [6]. In psoriasis, the involvement of JAKs has been shown and enabled the assessment of oral and topical JAK inhibitors as therapeutics. Tofacitinib, a pan-JAK inhibitor with predominant anti-JAK3 effect, has shown promising results in the treatment of psoriasis both orally [7] and topically [8]. Ruxolitinib, a JAK1/2 inhibitor used in the treatment of hematological diseases, has been tested in topical formulations to treat mild to moderate psoriasis, with favorable results [9]. However, the knowledge of the cutaneous JAK involvement in the ISDs is scarce and mostly based on *in vitro* or animal model analysis. In some of the most prevalent ISDs, such as mucosal lichen planus, cutaneous lupus erythematosus, atopic dermatitis and alopecia areata, Th1 and/or Th17 responses have been shown [10–16]. The main cytokines involved in Th1 and Th17 responses use JAKs for signaling [1, 17, 18]. Additionally, not only T cells, but also keratinocytes, dendritic cells, mast cells, eosinophils and macrophages could be activated [19, 20]. Due to the need of further elucidation of the JAK signaling in the ISDs, we aimed to analyze the cutaneous JAK/STAT expression in 6 prevalent ISDs. The set of ISDs comprises psoriasis, lichen planus (LP), cutaneous lupus erythematosus (CLE), atopic dermatitis (AD), alopecia areata (AA) and pyoderma gangrenosum (PG).

## Methods

### Human skin biopsies

Skin biopsies from patients with unequivocal clinical and histopathological diagnosis of psoriasis (n = 23), LP (n = 23; 8 cutaneous lichen planus, 9 lichen planopilaris, 6 mucous lichen planus), CLE (n = 22; 12 chronic discoid lupus, 6 subacute lupus, 1 acute lupus, 3 lupus tumidus), AD (n = 20), AA (n = 7), and PG (n = 10) were retrospectively collected from the Dermatology Department tissue biobank at the Ghent University Hospital, Belgium. Skin biopsies from healthy volunteers (n = 18) were used as controls. Additionally, lesional zskin biopsies from psoriasis patients (n = 10) and healthy controls (n = 10) were preserved in RNAlater solution (Life Technologies, Belgium) at -20°C. All skin biopsies included in this study were derived from patients who haven’t been receiving any systemic/topical immunosuppressant or immunomodulator during a minimum of 15 days before the excision. This study was carried out in accordance with the principles expressed in the Declaration of Helsinki. The protocol was approved by the Committee of Ethics of the Ghent University Hospital-Belgium (number: B670201318616/B670201318860). Written informed consent was obtained from the involved subjects before the skin biopsy was done for the qPCR analysis. Regarding the retrospective material, the patients were informed about the research and should contact the service only if they disagreed with the research.

### Immunohistochemistry

Biopsies were processed for immunohistochemistry and deparaffinized sections (4 μm) were stained using Dako immunohistochemistry products (Heverlee, Belgium). Antigens were retrieved using target retrieval solution (pH 6.1 or 9, 97°C) for 10–30 minutes. Slides were blocked using EnVision FLEX peroxidase-blocking reagent and incubated with the primary antibody during 1 h at room temperature. Detection was performed using the EnVision FLEX/HRP detection reagent and AEC+. After counterstaining using EnVision Flex Haematoxylin, slides were mounted and images were taken using Zeiss microscope (Axiophot), camera (Axiocam) and software (Axiovision) (Zeiss, Belgium).

The antibodies and their dilutions, pH and duration of antigen retrieval used, were consecutively: JAK2 (#3230, 1/50, pH9, 10’), pSTAT1 (#9167, 1/50, pH9, 10’) and pSTAT3 (#9145, 1/50, pH9, 10’), all rabbit monoclonal antibodies from Cell Signaling (Bioké, The Netherlands); JAK1 (ab47435, rabbit polyclonal, 1/200, pH9, 20’), JAK3 (ab61198, rabbit polyclonal, 1/50, pH9, 30’), TYK2 (ab115691, rabbit polyclonal, 1/50, pH6.1, 30’) and pJAK2 (ab32101, rabbit monoclonal, 1/200, pH9, 20’) (Abcam, United Kingdom); pJAK1 (sc-101716, 1/50, pH9, 30’) and pTYK2 (sc-11763, 1/50, pH6.1, 30’) rabbit polyclonal antibodies (Santa Cruz, Germany); pJAK3 (ABIN742868, 1/200, pH9, 30’) and pSTAT2 (bs-3428R, 1/100, pH9, 10’) rabbit polyclonal antibodies from Bioss Antibodies (Bio-connect, The Netherlands).

### Antibody validation

In order to validate the antibodies’ performance, positive control samples were included: breast carcinoma tissue for JAK1, JAK2, pJAK1, pSTAT2; cutaneous T cell lymphoma for JAK3, pJAK2 and pJAK3, Crohn’s disease and duodenum for respectively TYK2 and pTYK2, and psoriasis skin for pSTAT1 and pSTAT3 staining. Isotype controls were used to exclude antibodies which represented nonspecific binding.

### *In vitro* psoriasis model

To validate the epidermal staining profile, we used the monolayer *in vitro* psoriasis model described by Bracke *et al* using keratinocytes from a healthy donor.[21]. After isolated, the keratinocytes were cultured in 6 well culture plates containing keratinocyte growth medium (KGM, 2ml per wells) during 48h, when they reached at least 70% of confluence. Subsequently, the keratinocytes were divided in three groups and cultured during more 48h (medium was changed). Group 1 was induced to normal keratinocyte differentiation by culture in KGM and used as control; in group 2, psoriasis-associated features were induced by culture in KGM supplemented with 5% FBS (fetal bovine serum) and a pro-inflammatory cytokines mixture (10ng/ml IL-1α (Life Technologies, Belgium), 5 ng/mL IL-6, 5 ng/mL TNF-α, 10 ng/mL IL-17A [21] and 20 ng/mL IL-22 (all from Peprotech, United Kingdom)), and group 3 was induced to psoriasis-associated features as group 2 and treated with tofacitinib 1μM tofacitinib (Sigma-Aldrich, Belgium), which was added to the medium (simultaneously induction of psoriasis features and treatment with a JAK inhibitor). After 48 hours of induction, the cells were colleted, after removal of the medium and addition of trypsin EDTA, and fixed and embedded in paraffin.

Briefly, the cells were pelleted by centrifugation (400 rpm, 10’ at room temperature), fixed in 10% buffered formalin (Sigma-Aldrich, Belgium) during 2 h at room temperature and pelleted once more. Cell pellets were resuspended in 300 μL PBS and heated to 65°C in a warm water bath for less than 10 minutes. Next, 600 μL of melted agarose (3% low melting point agarose in PBS, at 65°C—Difco, United States) was added to the cells followed by immediate centrifugation. After gelation on ice, the gelled pellets were processed to paraffin blocks. Immunohistochemistry staining for pJAK1, pJAK2, pJAK3 and pTYK2 was done as described above.

### Staining analysis and classification

The 18 healthy controls and 20 psoriatic skin biopsies were stained with the complete JAK/STAT antibody panel. Regarding the other ISDs, samples of 10 patients (7 in the case of alopecia areata) were stained for JAK2, JAK3, TYK2, pJAK2, pJAK3, pTYK2. For JAK1, pJAK1 and the pSTATs, all the biopsies were stained (23 from LP, 22 from CLE, 20 from AD, 7 from AA and 10 from PG). Slides were blindly analyzed using Image J software.

In dermal inflammatory cells, the staining was classified as either ‘positive’ or ‘negative’. In the epidermal part, a distinction was made between cytoplasmatic and nuclear staining. When the staining was localized in the cytoplasm, the intensity and extent of staining were scored (extent 0: negative, 1: only basal layer, 2: ≤ ½ of the epidermal thickness and 3: > ½ of the epidermal thickness). For nuclear staining, the amount of stained cells per epidermal area (mm^2^) was counted.

### Statistical analysis

Statistical analysis was performed comparing each dermatosis with the healthy controls using SPSS Statistics 22. Differences between the groups were assessed by Kruskal-Wallis ANOVA for continuous variables. A Chi-Square test was used for the analysis of the not continuous variables. The results were considered statistically significant for p ≤ 0.05.

### RNA isolation and real-time qPCR analysis of skin biopsies

Untreated lesional psoriatic skin biopsies and healthy human skin obtained from patients undergoing plastic surgery were collected for JAK/STAT gene expression analysis. RNA isolation of the biopsies followed by RNA quality control and cDNA synthesis was done at Biogazelle (Biogazelle NV, Belgium). Briefly, the TissueLyser LT was used for the disruption and homogenization of the biopsies, prior to RNA extraction with the miRNeasy micro kit according to manufacturer’s recommendation (Qiagen, Belgium). RNA integrity was assessed by means of Experion automated electrophoresis system (Bio-Rad, Belgium). The software algorithm was used to calculate the RNA Quality Index (RQI) based on 28S/18S ribosomal RNA ratio. RNA concentrations were high (between 300 and 5500 ng/μL for a 15 μL eluate) and RNA integrity was acceptable (RQI: 6 to 9.3). The absence of gDNA was confirmed in all samples. cDNA synthesis was performed using the iScript Advanced cDNA Synthesis Kit (Bio-Rad, Belgium) with 800 ng as total RNA input. The cDNA quality of each sample was assessed by means of two human universally expressed genes. cDNA synthesis failed for one control sample which was excluded for further analysis. A geNorm pilot study was performed at Biogazelle to select proper, stably expressed reference genes for data normalization using a set of 8 candidate reference genes on all 19 samples. *B2M* and *PP1A* were identified as most stable reference genes. Therefore, the geometric mean of these two genes was used in order to calculate the optimal normalization factor needed for data normalization. qPCR-based gene expression analysis of *JAK1*, *JAK2*, *JAK3*, *TYK2*, *STAT1*, *STAT2*, *STAT3*, and the reference genes was done by use of validated PrimePCR assays (Bio-Rad, Belgium). All measurements were performed in 384-well plates in a reaction volume of 5 μL (with 5 ng cDNA input) using SYBR Green I. Each PCR was done in duplicate. Data-analysis was done in Biogazelle’s qBase+ software [22]. Due to the presence of high Cq values (> 30) or missing values for genes of interest, one psoriasis and two control samples were excluded for statistical analysis. Therefore, 9 psoriasis samples and 8 control samples were analyzed by the Mann-Whitney U test (2-tailed) in qBase+. The results were considered statistically significant for p ≤ 0.05.

## Results

In total 143 biopsies were analyzed (n = 123 immunohistochemistry; n = 20 qPCR analysis). The demographics for each disease group are summarized in Table 1.

**Table 1:** Demographics of the patients. The demographics for each disease group are comparable to the control group, except for AD.

	Age (mean)	P value(vs controls, Kruskal-Wallis ANOVA)	Men/women (%)	P value(vs controls, Chi-Square)
**Immunohistochemistry**				
Psoriasis (= 23)	49.1±19.5 years (range 17–90)	NS	47.8%/52.2%	NS
LP (n = 23)	49.5±14.9 years (range 23–73)	NS	34.8%/65.2%	NS
CLE (n = 22)	49±16 years (range 17–76)	NS	50%/50%	NS
AD (n = 20)	26.1±23.3 years (range 0.7–74)	p = 0.035	42.9%/57.1%	NS
AA (n = 07)	47±24.4years (range 15–82)	NS	50%/50%	NS
PG (n = 10)	56.2±18.5 years (range 34–84)	NS	40%/60%	NS
Controls (n = 18)	50.8±20 years (range 17–80)	/	38.9%/61.1%	/
**qPCR**				
Psoriasis (= 9)	49.2			
Controls (= 8)	52.9			

NS = not statistically significant. LP = lichen planus, CLE = cutaneous lupus erythematosus, AD = atopic dermatitis, AA = alopecia areata, PG = pyoderma gangrenosum.

### JAK/STAT expression in ISD skin biopsies

**Fig 1:**
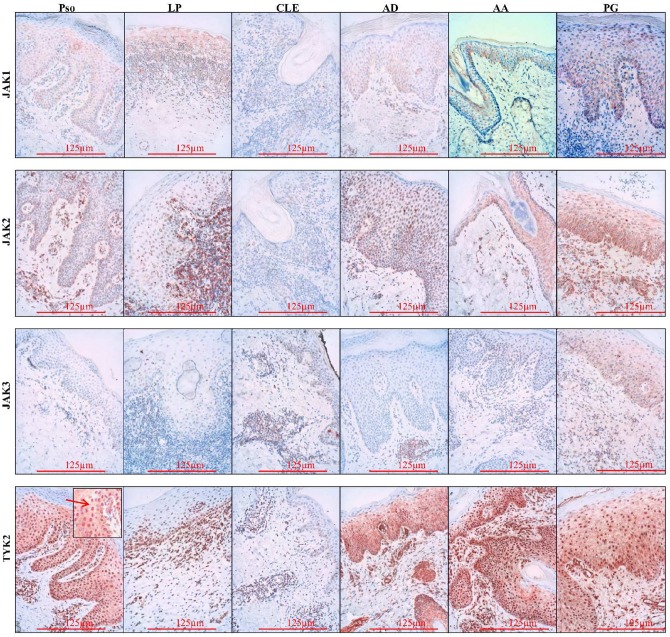
JAK1, JAK2, JAK3 and TYK2 immunohistochemical localization in the epidermis. Similar expression was seen in all studied diseases: JAK1, 2 and 3 were expressed in the cytoplasm of the keratinocytes and TYK2, besides cytoplasmic expression, had nuclear expression of TYK2 (arrow). Original magnification x 200. Pso = psoriasis, LP = lichen planus, CLE = cutaneous lupus erythemathosus, AD = atopic dermatitis, AA = alopecia areata, PG = pyoderma gangrenosum.

**Fig 2:**
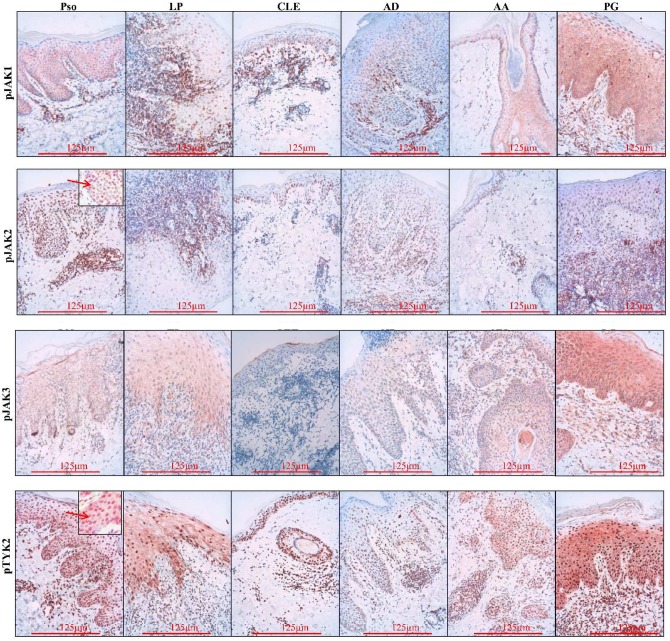
PhosphoJAK1, pJAK2, pJAK3 and pTYK2 immunohistochemical localization in the epidermis. Cytoplasmic expression of pJAK1, pJAK3 and pTYK2. Intranuclear expression of pJAK2 and pTYK2 (arrows). Similar expression was seen in all studied diseases. Original x 200. Pso = psoriasis, LP = lichen planus, CLE = cutaneous lupus erythemathosus, AD = atopic dermatitis, AA = alopecia areata, PG = pyoderma gangrenosum.

All 4 JAKs and their corresponding phosphorylated forms were positive in the epidermis and dermis of investigated diseases (Figs 1 and 2). They showed expression in the cytoplasm and along the cell membrane of keratinocytes, with the exception of pJAK2 which was only present in the nucleus of the cells (Fig 2 insert). In addition to the cytoplasmatic localization, TYK2 and pTYK2 were also expressed in the nucleus, with pTYK2 nuclear expression being more pronounced than the cytoplasmatic staining (Figs 1 and 2 insert). These data confirm previous reports on the intranuclear localization of pJAK2 and pTYK2, though their nuclear function remains to be clarified [23, 24]. Additional studies are necessary to clarify the intranuclear function of JAKs. Furthermore, in all the diseases, a perinuclear localization of JAK2, JAK3 and pJAK3 was observed in some of the slides. The cytoplasmic and nuclear localization of the pJAKs in the epidermis was confirmed in *in vitro* cultured keratinocytes.

As expected, pSTAT1, 2 and 3 are located into the nucleus of keratinocytes and/or dermal inflammatory cells, where they bind a DNA-recognition motif called gamma-activated sites (GAS) in the promoter region of cytokine-inducible genes and activate transcription [3].

### Various patterns of epidermal JAK/STAT expression in diseased versus healthy skin

**Fig 3:**
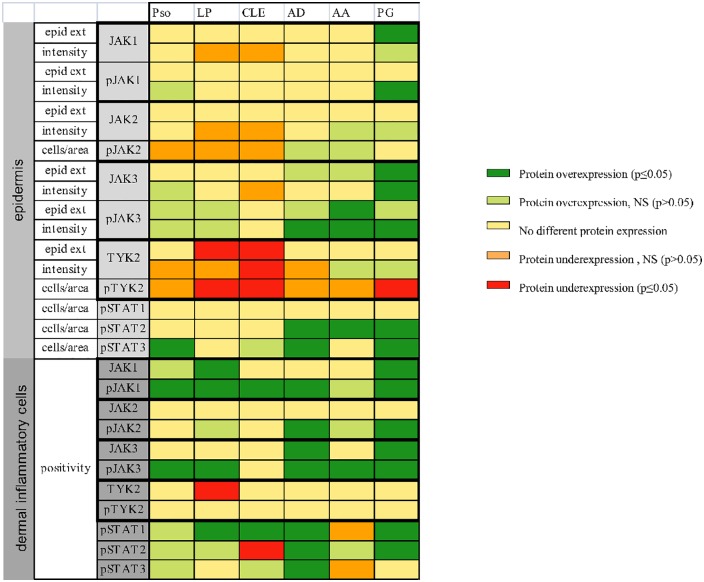
Overview of JAK/STAT protein (by immunohistochemistry) expression in the studied inflammatory skin diseases as compared to healthy skin. Pso = psoriasis, LP = lichen planus, CLE = cutaneous lupus erythemathosus, AD = atopic dermatitis, AA = alopecia areata, PG = pyoderma gangrenosum, epid ext = epidermal extent. NS = not statistically significant.

**Fig 4:**
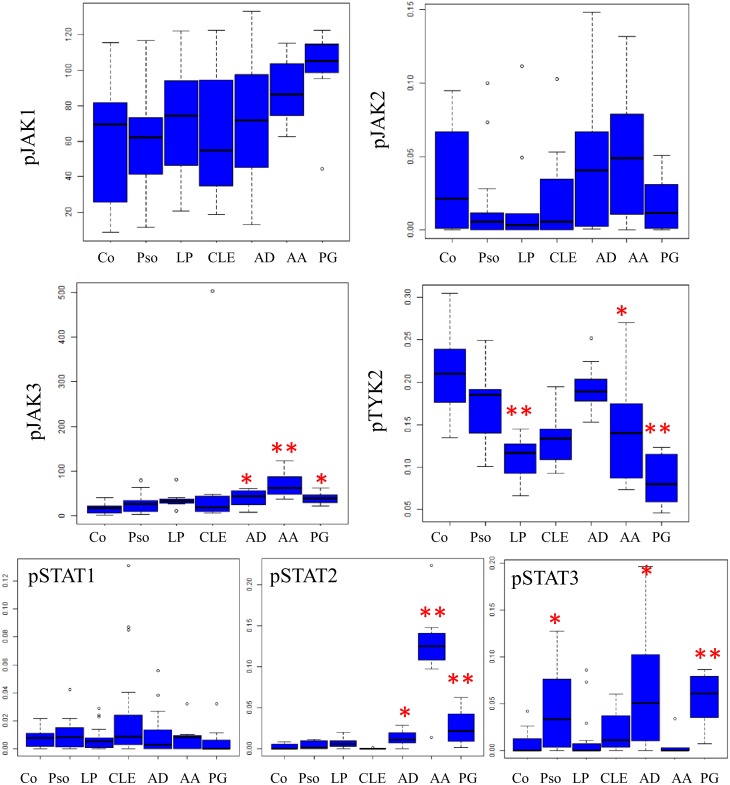
SPSS statistical analysis of the keratinocyte staining compared with control (staining intensity for pJAK1 and pJAK3 and density of the positively stained cells for pJAK2, pTYK2, pSTAT1, pSTAT2 and pSTAT3). Pso = psoriasis, LP = lichen planus, CLE = cutaneous lupus erythemathosus, AD = atopic dermatitis, AA = alopecia areata, PG = pyoderma gangrenosum, epid ext = epidermal extent. NS = not statistically significant. * p≤0.05 ** p≤0.001.

In the epidermis, JAK3 but mainly it phosphorylated form showed a higher expression in all ISDs except in CLE (p value for the pJAK3 intensity analysis for AD, AA and PG was, respectively: 0.018; < 0.001 and 0.018). JAK1 and pJAK1 were only significantly overexpressed in PG (p = 0.013 for JAK1, p = 0.024 for pJAK1). No significantly different expression was seen for JAK2 and pJAK2 (Figs 3 and 4) in any of the investigated ISDs. Conversely, TYK2 and pTYK2 was underexpressed in the epidermis of psoriasis, LP, CLE and PG (Figs 3 and 4). In AA and PG, TYK2 epidermal intensity was higher compared to control skin, although pTYK2 was underexpressed (Figs 3 and 4).

Strong pSTAT2 overexpression was present in AD, AA and PG (p = 0.03, < 0.001 and 0.009 respectively) and pSTAT3 in psoriasis, AD and PG (p = 0.034, 0.002 and 0.001 respectively) while there was no obvious pSTAT1 signal in de epidermal layer (Fig 4). Thus, LP shows no differences in epidermal pSTAT expression compared to healthy skin. Moreover, there was no increased epidermal expression of any JAK/STAT member in lupus, except for a slight increase in pSTAT3. Even after excluding lupus tumidus, a dermal form of lupus, the results remained unchanged.

### Different expression of JAK/STAT members in dermal inflammatory cells

Compared to healthy skin, the expression of both pJAK1 (p-value Pso: 0.002, LP: 0.01, CLE: < 0.001, AD: 0.002, PG: 0.004) and pJAK3 (p-value Pso: 0.003, LP: 0.001, AD: < 0.001, AA: 0.002, PG: < 0.001) was significantly enhanced in the dermal inflammatory cells of most studied diseases (Fig 3, Table 2). This overexpression was more pronounced than in the epidermal keratinocytes. The expression of (p)TYK2 in the inflammatory cells of ISDs was comparable to that of healthy skin, with a significant underexpression of TYK2 in LP (p < 0.001). None of the ISDs is characterized by an increased expression of JAK2, however pJAK2 was overexpressed in AD (p = 0.042) and PG (p = 0.009). Phospho-STAT expression in dermal inflammatory cells was highly dependent on the type of ISD (Fig 3, Table 2).

**Table 2:** Percentage of JAK/STAT family positivity (in grey)/negativity in dermal inflammatory cells of ISDs comparing to controls.

		Controls	Pso	LP	CLE	AD	AA	PG
**JAK1**	Positive(%)	26(4/15)	64(9/14)	61*(13/21)	50(10/20)	50(10/20)	42(3/7)	80*(8/10)
	Negative(%)	74(11/15)	36(5/14)	29(8/21)	50(10/20)	50(10/20)	68(4/7)	20(2/10)
**pJAK1**	Positive(%)	43.75(7/16)	94,7*(18/19)	85,71*(18/21)	100*(20/20)	95*(19/20)	71.4(5/7)	100*(10/10)
	Negative(%)	56.25(9/16)	5.26%(1/19)	14.29(3/21)	0	5(1/20)	28.6(2/7)	0
**JAK2**	Positive(%)	100(18/18)	100(20/20)	100(10/10)	100(10/10)	100(10/10)	85(6/7)	100(10/10)
	Negative(%)	0	0	0	0	0	15(1/7)	0
**pJAK2**	Positive(%)	47(8/17)	58.8(10/17)	80(8/10)	60(6/10)	90*(9/10)	71(5/7)	100*(10/10)
	Negative(%)	53(9/17)	41.2(7/107)	20(2/10)	40(4/10)	10 (01/10)	29(2/7)	0
**JAK3**	Positive(%)	11(2/18)	13(2/15)	10(1/10)	40(4/10)	70*(7/10)	14.3(1/7)	90*(9/10)
	Negative(%)	89(16/18)	87(13/15)	90(9/10)	60(6/10)	30(3/10)	85.7(6/7)	10(1/10)
**pJAK3**	Positive(%)	5,56(1/18)	52,63*(10/19)	70*(7/10)	20(2/10)	80*(9/10)	71.4*(5/7)	80*(8/10)
	Negative(%)	94,44(17/18)	47,37(9/19)	30(3/10)	80(8/10)	20(1/10)	28.6(2/7)	20(2/10)
**TYK2**	Positive(%)	100(17/17)	100(19/19)	20*(2/10)	100(10/10)	100(10/10)	100(7/7)	100(10/10)
	Negative(%)	0	0	80(8/10)	0	0	0	0
**pTYK2**	Positive(%)	94(17/18)	83(15/18)	100(10/10)	90(9/10)	100(10/10)	100(7/7)	100(10/10)
		6(1/18)	17(3/18)	0	10(1/10)	0	0	0
**pSTAT1**	Positive(%)	12,5(2/16)	41(7/17)	59*(13/22)	84.2*(16/19)	50*(10/20)	0	80*(8/10)
	Negative(%)	87.5(14/16)	59(10/17)	41(9/22)	15.8(3/19)	50(10/20)	100(7/7)	20(2/10)
**pSTAT2**	Positive(%)	29.4(5/17)	55(10/18)	47.6(11/21)	0	85*(17/20)	71(5/7)	100*(10/10)
	Negative(%)	70.6(12/17)	45(8/18)	52.4(10/21)	100(20/20)	15(3/20)	29(2/7)	0
**pSTAT3**	Positive(%)	43.7(7/16)	62.5(10/16)	50(10/20)	65(13/20)	80*(16/20)	14.2(1/7)	40(4/10)
	Negative(%)	56,3(9/16)	37.5(6/16)	50(10/20)	35(7/20)	20(4/20)	85.8(6/7)	60(6/10)

Pso = psoriasis, LP = lichen planus, CLE = cutaneous lupus erythematosus, AD = atopic dermatitis, AA = alopecia areata, PG = pyoderma gangrenosum,

* = significant expression.

### qPCR analysis of JAK/STAT expression in psoriasis confirms *JAK3* overexpression

**Fig 5:**
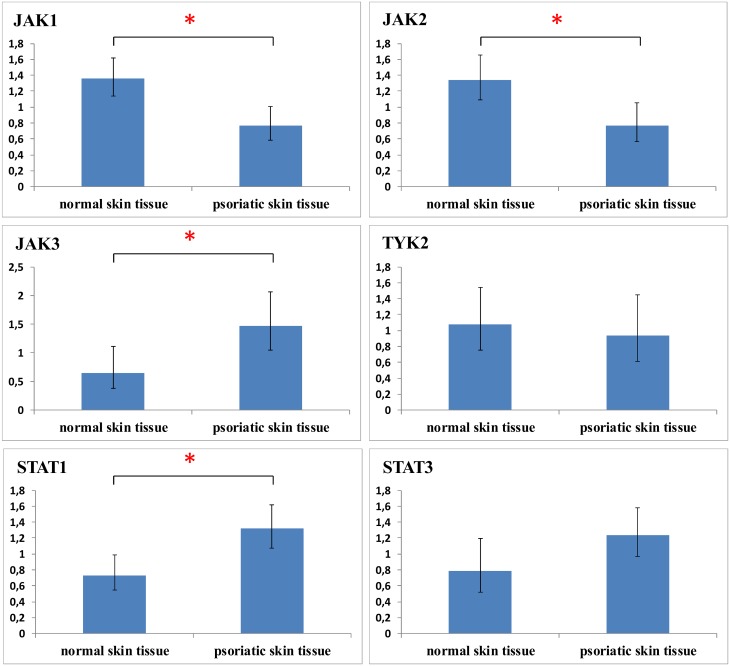
JAK/STAT mRNA levels in human skin biopsies from psoriasis compared to healthy controls illustrates *JAK3*, *STAT1* and *STAT3* overexpression. * p≤0.05.

The expression of JAK/STATs in psoriatic skin was further elucidated by qPCR (Fig 5). Compared to healthy skin, *JAK3* (p = 0.024) was significantly overexpressed, while *JAK2* (p = 0.001) and *TYK2* (p = 0.604) exhibited a decreased expression. These results are in agreement with the JAK immunohistochemistry expression profile (Fig 3). Overall, the *JAK1* expression in psoriatic skin was lower than healthy controls (p = 0.02). Regarding STATs, qPCR results indicated overexpression of *STAT3* (p = 0.052) and *STAT1* (p = 0.005) in psoriasis (Fig 5).

### Stimulation and inhibition experiments

**Fig 6:**
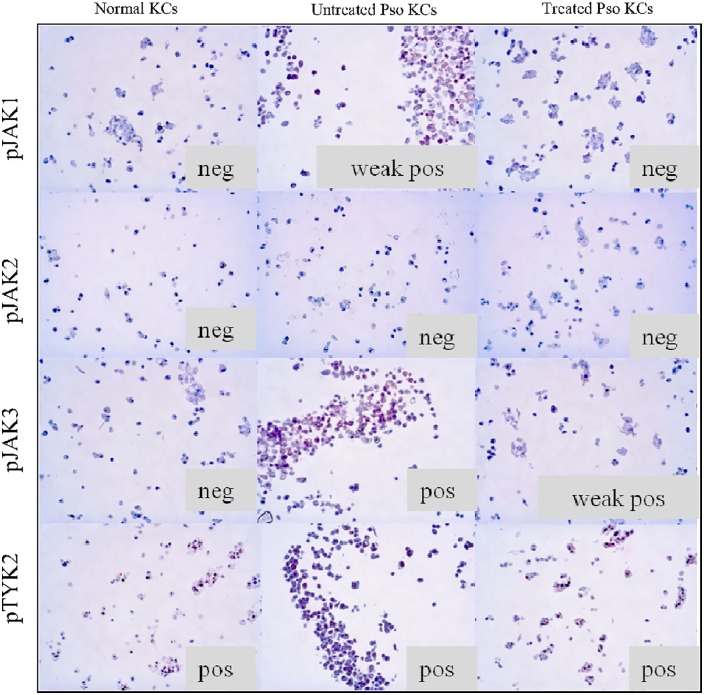
Immunohistochemical staining of normal keratinocytes (KCs) and psoriasis induced keratinocytes (Pso KCs) with or without tofacitinib treatment. The pJAKs expression in Pso KCs was similar and summarizes the one observed in the epidermis of the psoriasis skin biopsies. Strong pJAK3 expression and weak positive pJAK1 expression was induced by psoriasis stimulation and inhibited after treatment with tofacitinib. Phospho-JAK2 and pTYK2 expression did not change neither with psoriasis stimulation nor with the treatment. Note the cytoplasm localization of pJAK1 and pJAK3 and the nuclear localization of pTYK2 in the keratinocytes. As pJAK2 was negative in all conditions, the localization of the staining could not be analysed. Original magnification x200.

We used our previously developed *in vitro* psoriasis model [21] to confirm the expression patterns observed in the psoriatic biopsies. A psoriatic phenotype was induced in keratinocytes as previously described by our group (Fig 6). A marked pJAK3 overexpression was found with a less intense pJAK1 induction corresponding to the psoriasis biopsy analysis. A subsequent treatment with tofacitinib showed reversal of pJAK3 and pJAK1 expression demonstrating a direct interaction of JAK inhibitors with psoriatic keratinocytes.

## Discussion

To our knowledge, this is the first study investigating the expression of the JAK/STAT components in a panel of inflammatory skin diseases using human skin disease material. Overall, the JAK3 pathway was most upregulated followed by JAK1 and to a lesser extent JAK2. In the epidermal layer, JAK3 was overexpressed in all ISDs except CLE indicating its crucial role in inflammatory keratinocytes. This suggests that JAK3 inhibition is the most promising approach for topical application as it can directly reverse the JAK profile of keratinocytes. In our in vitro psoriasis model we showed that tofacitinib directly inhibits pJAK3 and pJAK1 expression in psoriatic keratinocytes. This implies that topical treatment with tofacitinib may exert a direct impact on the psoriatic phenotype independent of its activities on infiltrating immune cells. This indicates that deep dermal infiltration of topical JAK inhibitors, increasing the likelihood of systemic absorption, might not be necessary.

Downstream, pSTAT2 and pSTAT3 are strongly expressed while no signs of STAT1 activation were found. The importance of STAT3 in inflammation has already been demonstrated. It has been proven that STAT3 plays a role in keratinocyte differentiation and proliferation, is activated in psoriatic lesions [25–27] and is a therapeutic target for the treatment of psoriasis [27]. This suggests that topical application of JAK inhibitors would mainly act on the STAT3 cascade. The role of STAT2 is still unknown, but it has been suggested that STAT2 plays a role in the activation of STAT1 and STAT3 [28]. In the dermal layer, the inflammatory immune cells in most ISDs showed expression of all JAKs, with the exception of TYK2, although the subsequent STAT signaling was dependent on the type of skin disease.

Our results indicate that psoriasis is mainly a JAK3 and JAK1 driven disease with a predominance of STAT3 signaling. *STAT3* upregulation was seen in our qPCR analysis of psoriatic samples. These findings corroborate with previous reports [26]. It has been shown that STAT3 is involved in the upregulation of keratin 17, a hallmark of psoriasis, in keratinocytes following IL-17A stimulation [29]. *In vitro*, JAK3/STAT3 signaling acts as a target in the treatment of psoriasis models [30, 31]. Tofacitinib, one of the most investigated JAK inhibitors in the treatment of psoriasis, shows impressive beneficial responses with JAK3 as their predominant target [7, 8]. Nonetheless, ruxolitinib, a JAK1/2 inhibitor, has also proven its efficacy [9].

AA and LP also displayed a strong JAK3 expression. In AA, JAK1 and JAK2 were to a lesser extent also elevated. The resulting downstream cascade seems based on the expression levels mainly conducted by pSTAT2. This is remarkable as AA is considered to be a IFN-γ driven disease which would result in JAK1, JAK2 and STAT1 activation. In this regard, it is important to notice that the landmark paper of Xing *et al* [32] did also found JAK3 as the only JAK overexpressed in human AA compared to controls. Moreover, in their mice experiments JAK3 inhibition also slightly outperformed JAK1/JAK2 antagonism. In a case report, a patient receiving tofacitinib for psoriasis experienced remission of his longstanding alopecia universalis [33]. Currently, there are phase II trials ongoing to assess tofacitinib in alopecia areata (NCT02197455, NCT02553330).

Little is known about JAK expression in LP. Nonetheless, the JAK3 and JAK1 overexpression which was especially present in the inflammatory infiltrate indicate that JAK1/3 inhibition might be a successful approach in LP. In CLE, only a JAK1 overexpression was found in the dermis. This is in accordance with the results of Li *et al* involving patients with systemic lupus erythematosus displaying pSTAT1 protein activation in their inflammatory malar rash [34].

In AD an PG, all three JAKs and STATs were overexpressed. As PG is a highly inflammatory disorder this might not be surprising. In AD, JAK research is still in its early phase. Nonetheless, murine experiments and a small human trial demonstrated the potential of tofacitinib in recalcitrant atopic dermatitis [35, 36]. In atopic dermatitis, IL-4 is the main involved interleukin [20] and may phosphorylate different JAKs in several cell types [37]. In human B lymphocytes, IL-4 induces phosphorylation of JAK1 and JAK3 [38].

In the studied diseases, JAK2 did not emerge as pivotal therapy target as pJAK2 was only overexpressed in the inflammatory dermal cells of AD and PG. Regarding the systemic JAK inhibitors, analysis of clinical trials using JAK inhibition in the treatment of immune mediated inflammatory diseases attributed hematological side effects to JAK2 blockade and suggested selective JAK1/3 inhibition to increase efficacy and safety [39]. Our results indicate that the role of JAK2 in ISD pathophysiology is probably limited, considering the low expression of JAK2 in ISDs.

A striking reduced expression of TYK2 and pTYK2 was found at the epidermal level. The importance of TYK2 in inflammatory diseases is based on genetic polymorphisms and murine experiments with knockout mice demonstrating its role in IL22 and IL23-mediated signaling in psoriasis [40]. However, increased TYK2 expression has been scarcely reported and TYK2 downregulated mRNA has been previously observed in psoriasis [26]. This suggests a complex regulation of TYK2 *in vivo*.

This study of JAK/STAT expression in some prevalent ISDs was retrospective, what prevented the collection of all clinical characteristics of the patients, and had a small sample size. Nevertheless, there was a comparable population among the groups (except for AD, which affects younger patients) and the immunohistochemical findings were validated by qPCR for psoriasis. Moreover, we conducted an extensive validation of the used antibodies by cell stimulation and inhibition experiments.

## Conclusion

The development of alternative strategies in the treatment of ISDs is desirable, as there is an unmet therapeutic need. In this context JAK blockers have emerged as a promising option for topical or systemic therapy. The topical formulation makes these drugs more attractive for dermatological purposes as they offer an improved safety profile. Our study evidenced that pan-JAK inhibitors seem the most appropriate approach in PG and AD, based on the JAK expression levels. In contrast, AA, psoriasis and LP carry mainly a JAK3 signature which suggests that specific JAK3 inhibition could be sufficient. In CLE, further research on the efficacy of JAK1 inhibitors is warranted. Considering the epidermal expression levels, our results show most evidence for topical JAK3 inhibitors in ISDs.
